# The relationship between Chinese medical students' learning motivation and learning engagement: a network analysis

**DOI:** 10.3389/fpsyg.2025.1611900

**Published:** 2025-10-14

**Authors:** Dengqin Wang, Peibo Song, Qianqian Zhang, Huili Zhao, Bo Zhuang

**Affiliations:** ^1^Medical Comprehensive Training Center, Jining Medical University, Jining, China; ^2^School of Mathematics, Shandong University, Jinan, China; ^3^Clinical Medical College, Jining Medical University, Jining, China

**Keywords:** medical students, learning motivation, learning engagement, network analysis, intrinsic motivation, extrinsic motivation

## Abstract

**Objective:**

This study aims to elucidate the internal relationships among the dimensions of learning motivation and learning engagement in Chinese medical students by examining the network structure of these constructs and exploring potential differences under different training models.

**Methods:**

A network analysis approach was adopted to analyze survey data from 499 Chinese medical students. A comprehensive network encompassing dimensions of learning motivation and learning engagement was constructed, followed by two sub-networks respectively focusing on tuition-exempt (government-funded) medical students and self-funded medical students. Comparisons were then made to highlight structural differences across these sub-networks.

**Results:**

In the overall network, “challenge” had the highest expected influence index, followed by “focusing on interpersonal competition.” These two dimensions formed the central hub of the network and exerted significant influence on the network's overall structure. Comparative analyses showed that in the government-funded student network, “focusing on interpersonal competition” exhibited the highest expected influence, whereas “challenge” was most influential in the self-funded student network.

**Conclusion:**

This study offers a novel perspective for understanding medical students' learning motivation and behavioral patterns, thereby expanding research avenues in the field of medical education. The findings suggest that targeted interventions on these core dimensions can effectively enhance learning engagement among medical students.

## 1 Introduction

In medical education, learning motivation and learning engagement are tightly interwoven and mutually influential. However, prior studies often aggregate dimensions into total scores or specify directional paths among composite variables, thereby obscuring which dimensions are structurally central or serve to connect motivation and engagement (Wu et al., 2020; Bin et al., 2023). Little is known about how the dimensions of learning motivation interact with facets of engagement among Chinese undergraduate medical students, or whether these structural relations differ between publicly funded and self– funded training models. To address this gap, we employ psychological network analysis to map conditional associations among dimensions, identify hub and bridge nodes, and compare networks across groups to inform targeted instructional strategies.

Learning engagement refers to the time and effort individuals invest in educationally purposeful activities (Kahu, 2013), manifested in the array of student-related learning endeavors (Astin, 1999). As a key indicator in positive psychology, it enhances students' development and boosts their interest in learning (Heng, 2014). Previous studies have shown that learning engagement exerts a positive effect on learning outcomes, academic achievements, and the mitigation of academic burnout among college students (Kassab et al., 2024; Lei et al., 2018; Zhao et al., 2021). For medical students, learning engagement not only influences the quality of medical education but also bears on the future development of the healthcare sector. However, research indicates that Chinese medical undergraduates generally report moderate levels of learning engagement (Chen et al., 2022; Li F. et al., 2019; Tao et al., 2025), and in some cases even lower than the midpoint (Shen et al., 2021). Consequently, it is necessary to investigate factors influencing medical students' learning engagement and the mechanisms through which these factors operate.

Learning motivation is among the core driving forces behind students' engagement. It ignites and sustains students' learning goals and behaviors (Cook and Artino, 2016). In recent years, research on college students' learning motivation has become a focal area in educational psychology (Liu et al., 2012; Wouters et al., 2017). Studies suggest that intrinsic motivation is pivotal for fostering students' active participation in the learning process (Oudeyer et al., 2016). Intrinsic motivation arises from students' inherent interest in and satisfaction derived from a learning activity itself. When students find enjoyment or perceive meaning in learning, they are more inclined to invest time and effort, actively engage in discussions, complete assignments, and explore knowledge in depth (Flannery, 2017). In contrast, extrinsic motivation relies on external rewards or pressures, such as grades, scholarships, or social recognition. Although extrinsic motivation can stimulate learning behaviors to some extent, it tends to be less enduring and profound than intrinsic motivation.

Research by Wu et al. (2020) highlights a close association among learning motivation, self-efficacy, and engagement, revealing a significant positive relationship among these factors. Bin et al. (2023) have similarly demonstrated that medical students' learning motivation correlates significantly with self-efficacy, learning engagement, and satisfaction with academic outcomes. Notably, the relationship between learning motivation and student engagement is not unidirectional but reciprocal. On one hand, high levels of learning motivation promote active student participation and lead to improved academic performance and learning satisfaction; on the other hand, positive experiences of engagement reinforce students' motivation. Despite ample research on the relationship between learning motivation and student engagement, several key questions remain. Specifically, how do different dimensions of learning motivation and engagement interact? Which dimensions are most central? An in-depth examination of these core dimensions and their interrelations not only clarifies the complex interplay between motivation and engagement but also provides a scientific basis for effective interventions aimed at enhancing medical students' learning engagement.

Network analysis offers a systematic method for examining the internal structure of psychological constructs through visualization (Burger et al., 2023; Epskamp et al., 2018; Zhu et al., 2023). By viewing the variables within a construct and their interrelationships as an integrated network, the approach analyzes the patterns of connections (e.g., identifying central nodes and key pathways) to reveal core features and underlying mechanisms (Borgatti et al., 2009; Guloksuz et al., 2017). Identifying central nodes is especially crucial: it can illuminate how a construct is organized internally (Briganti et al., 2018; Dalege et al., 2016) and guide the design of targeted interventions. By intervening in these central nodes, the entire construct can be more effectively influenced (Borsboom and Cramer, 2013). Currently, network analysis has been widely applied in mental health and psychology, yielding significant findings (Funkhouser et al., 2021; Liang et al., 2022; Santos et al., 2018). In recent years, this method has also been introduced to the educational domain, providing new perspectives and tools for educational psychology research (Kwon et al., 2024; Li Y. et al., 2024; Wang et al., 2025). Guided by current recommendations for reporting and interpreting cross-sectional psychological networks, we estimated a regularized partial-correlation network to identify nodes with high expected influence (EI) that may serve as levers for intervention. Unlike traditional bivariate correlations or omnibus regression approaches, our method retains the sign of associations, models the simultaneous dependencies among all dimensions, and can quantify invariance in structure and strength across groups. Whereas many prior educational applications stop at visualization or global centrality summaries, we extend this by translating central nodes into actionable targets for instructional intervention.

Globally, inequitable distribution of healthcare resources is a prevalent issue, prompting many countries to adopt economic incentives to attract medical graduates to rural areas (Abid et al., 2020; Matsumoto et al., 2021; Rabinowitz, 1988). China faces similar challenges of uneven healthcare development, particularly the shortage of grassroots medical professionals (Deng et al., 2023; Guo et al., 2019). In response, the government initiated a rural “order-oriented tuition-exempt medical student” (hereafter “government-funded medical student”) training program in 2010 (Notice on Issuing the Implementation Opinions on Conducting Free Training for Rural Order-Oriented Medical Students, n.d.). Through policies such as tuition waivers, free housing, and living allowances, this program aims to cultivate medical professionals committed to serving in grassroots communities, requiring them to fulfill a six-year service contract at designated local healthcare institutions upon graduation.

Against this backdrop, the present study employs a network analysis approach to comprehensively investigate the core dimensions of learning motivation and learning engagement among medical students and elucidate the underlying mechanisms of their interconnections. By constructing network models, this study not only examines the interactions among various dimensions but also identifies critical factors influencing learning engagement. In addition, comparative analyses of students under different training models are conducted to uncover distinctive learning characteristics, thereby providing evidence-based insights for optimizing medical student training programs and developing targeted educational interventions.

## 2 Methods

### 2.1 Participants

We invited all enrolled third–year undergraduate students at the target medical school to participate via class channels (hyperlink/QR code). Eligibility required current third–year status and completion of all questionnaire items; participation was voluntary and anonymous. Relevant studies suggest that for networks with fewer than 20 nodes, a minimum sample size should generally exceed 20 times the number of nodes or comprise at least 250–350 cases (Constantin and Cramer, 2018). Moreover, whether a given sample affords sufficient statistical power in network analysis should be judged alongside stability parameters (Constantin et al., 2023). We distributed 540 questionnaires and analyzed 499 valid responses (see Table 1 for gender, place of origin, academic rank, and parental education), meeting the minimum sample requirements. We focused on third–year students because their career planning and learning goals are typically more defined at this stage, which reduces heterogeneity in motivational orientations and allows a clearer depiction of engagement. This study was approved by the Ethics Committee of Jining Medical University. Clinical trial number: not applicable.

**Table 1:** General demographic characteristics of participants.

**Characteristics**	**All(*n* = 499)**	**Government-funded medical students (*n* = 248)**	**Non-government-funded medical students (*n* = 251)**
**Sex**			
Male	245(49.1)	131(52.8)	114(45.4)
Female	254(50.9)	117(47.2)	137(54.6)
**Place of origin**			
Urban	229(45.9)	86(34.7)	143(57.0)
Rural	270(54.1)	162(65.3)	108(43.0)
**Academic performance**			
Top 50% in grade ranking	302(60.5)	140(56.5)	162(64.5)
Bottom 50% in grade ranking	197(39.5)	108(43.5)	89(35.5)
**Father's education level**			
Junior high school or below	204(40.9)	99(39.9)	105(41.8)
Senior high school or technical secondary school	146(29.3)	83(33.5)	63(25.1)
Junior college	67(13.4)	28(11.3)	39(15.5)
Bachelor's degree or above	82(16.4)	38(15.3)	44(17.5)
**Mother's education level**			
Junior high school or below	262(52.5)	140(56.5)	122(48.6)
Senior high school or technical secondary school	133(26.7)	70(28.2)	63(25.1)
Junior college	61(12.2)	27(10.9)	34(13.6)
Bachelor's degree or above	43(8.6)	11(4.4)	32(12.7)

### 2.2 Learning motivation scale

This study used the Chinese version of the learning motivation scale developed by Amabile et al. and adapted by Chi and Xin (2006). The scale comprises 30 items distributed across six dimensions: challenge (8 items), enthusiasm (6 items), dependence on others' evaluations (6 items), choosing simple tasks (4 items), focusing on interpersonal competition (4 items), and pursuit of rewards (2 items). The “challenge” and “involvement” dimensions constitute the intrinsic motivation subscale, whereas “reliance on others” “evaluations,” “preference for simple tasks,” “focusing on interpersonal competition,” and “pursuit of rewards” constitute the extrinsic motivation subscale. Each item is rated on a 4-point Likert scale, from 1 (“completely inconsistent”) to 4 (“completely consistent”), with higher scores indicating stronger learning motivation. In this study, the overall Cronbach's α of the scale was 0.874.

### 2.3 Learning engagement scale

This study employed a modified version of the Utrecht Work Engagement Scale–Student (UWES-S) developed by Dong (2021). The scale comprises 15 items across three dimensions: vitality (3 items), dedication (3 items), and behavior (9 items). Each item is rated on a 5-point Likert scale, from 1 (“completely inconsistent”) to 5 (“completely consistent”), with higher scores indicating higher levels of learning engagement. In this study, the overall Cronbach's α of the scale was 0.906.

### 2.4 Data analysis

Data analysis involved descriptive statistics and network analysis. First, SPSS 24.0 was used for descriptive statistics to explore participants' basic information and relevant demographic variables. Second, R 4.4.2 was employed for network analysis to investigate the structural relationships among the dimensions of learning motivation and learning engagement. The network analysis included the following five components: network estimation, network visualization, centrality estimation, network comparison, and assessments of network accuracy and stability.

#### 2.4.1 Network estimation

Following the standardized procedures proposed by Epskamp and Fried (2018), we used the qgraph package in R to estimate partial correlation networks for the overall sample and for two subgroups (government-funded vs self-funded medical students). In the resulting network graphs, circular nodes represent each dimension, and the lines between them (edges) indicate partial correlations, with line thickness indicating the magnitude of the partial correlation coefficient. Specifically, a Gaussian Graphical Model (GGM) was first used to obtain a preliminary estimation of the network structure. Subsequently, the Least Absolute Shrinkage and Selection Operator (LASSO) was applied to regularize the model and shrink smaller edges to zero, thereby reducing false positives and enabling more precise identification of the underlying network structure (van Borkulo et al., 2014).

#### 2.4.2 Network visualization

Network visualization was performed for three sets of data: the overall network of medical students' learning motivation and engagement, the network of government-funded medical students, and the network of self-funded medical students. All networks were visualized using the Fruchterman–Reingold algorithm. Blue edges represent positive correlations, and red edges represent negative correlations; thicker lines signify stronger connections between nodes.

#### 2.4.3 Centrality estimation

This study used the Expected Influence (EI) index as the core indicator of a node's influence within the network. EI is defined as the sum of the edge weights connected to a given node. A higher EI value indicates greater influence in the network. Unlike traditional measures of centrality (e.g., strength centrality), EI retains the original sign of the edge weights rather than taking their absolute values, enabling differentiation of positive and negative associations between nodes and providing a more comprehensive view of each node's impact within the network (Robinaugh et al., 2016).

#### 2.4.4 Network comparison

We employed the Network Comparison Test package in R to conduct global and local invariance tests at a significance level of 0.05 (van Borkulo et al., 2023). If the p-value is below 0.05, the networks are considered significantly different. The global invariance test consists of two components: (1) Network Structure Invariance, which assesses structural similarities by comparing the maximum absolute difference in edge weights across networks; and (2) Global Strength Invariance, which evaluates differences in overall network strength by comparing the sum of all absolute edge weights. Local invariance tests further compare individual edge weights and node centrality indices between networks, with adjustments made using the Holm–Bonferroni method.

#### 2.4.5 Network Accuracy and Stability

We used the bootnet package to estimate network accuracy and stability. A 1,000-iteration nonparametric bootstrap was conducted to generate 95% confidence intervals (CIs) for edge weights, where narrower intervals indicate higher accuracy. The centrality stability coefficient (CS) was used to evaluate network stability, with CS > 0.25 indicating acceptable stability and CS >0.50 signifying good stability. Because the data derive from self-report Likert-type responses, we acknowledge potential social-desirability bias and ceiling effects. We emphasized confidentiality and anonymity to reduce evaluation apprehension; nevertheless, we interpret the associations cautiously and recommend that future work incorporate multimethod designs (e.g., behavioral traces, observation) to complement self-reports.

## 3 Results

### 3.1 Descriptive statistics

Table 1 presents the general demographic characteristics of the participants. The results indicate that among government-funded medical students, the proportion of males (52.8%) exceeded that of females (47.2%). Moreover, the majority of students (54.1%) came from towns or villages, with rural-origin students comprising a significantly larger share of the government-funded group (65.3%) compared to those from urban areas (43.0%). In terms of academic performance, 60.5% ranked in the top 50% of their cohort. Additionally, most fathers and mothers had a junior high school education or below, at 40.9% and 52.5%, respectively.

### 3.2 Network estimation of medical students' learning motivation and learning engagement

**Figure 1:**
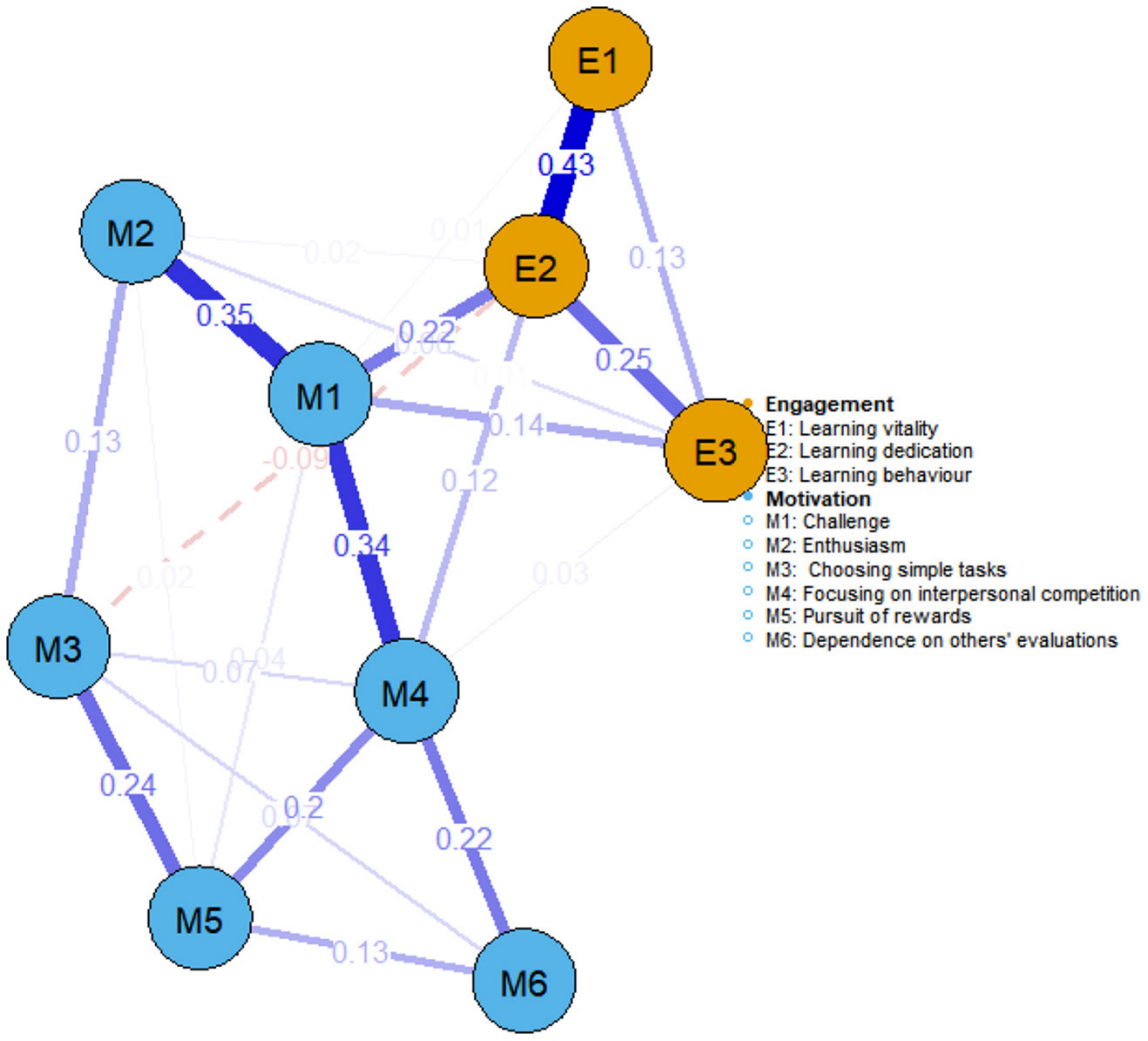
Learning motivation and learning engagement network for the overall sample (*n* = 499). Note: Each node represents a dimension. Blue nodes denote six dimensions of learning motivation, and yellow nodes denote three dimensions of learning engagement. The edges connecting two nodes represent partial correlations; thicker edges indicate stronger partial correlations, and thinner edges indicate weaker partial correlations. Blue edges signify positive correlations, while red dashed edges signify negative correlations.

As depicted in Figure 1, the overall sample network—comprising nine nodes and 23 edges—had a mean edge weight of 0.087 and a density of 0.639. The learning motivation nodes and learning engagement nodes formed two distinct clusters. The primary link connecting learning motivation and learning engagement was between challenge and learning dedication, showing the strongest correlation (weight = 0.22). Within the learning motivation cluster, challenge was closely associated with both enthusiasm and focusing on interpersonal competition (weights = 0.35 and 0.34, respectively). Within the learning engagement cluster, learning dedication was strongly linked to learning vitality (weights = 0.43).

**Figure 2:**
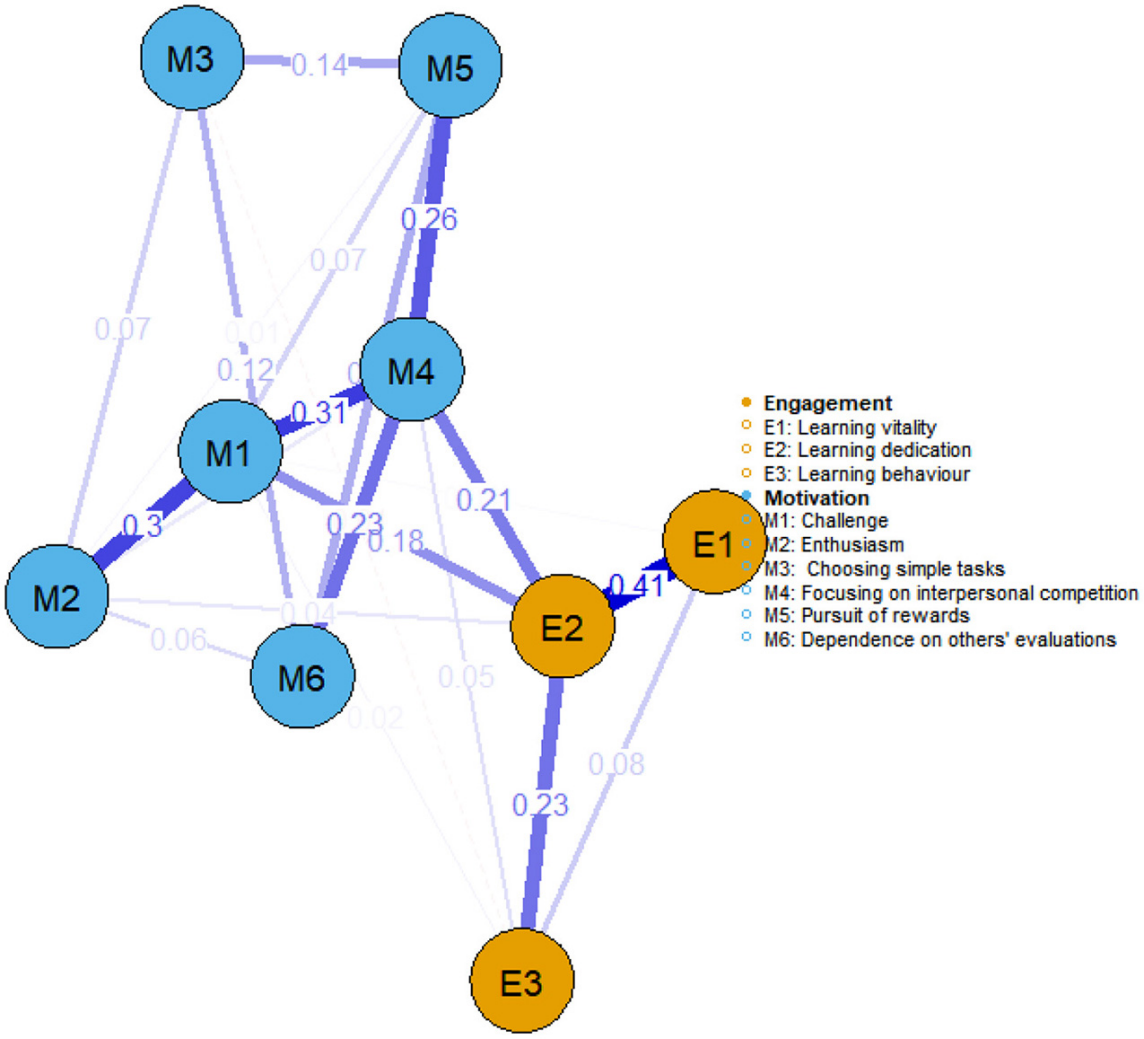
Network structure of learning motivation and learning engagement among government-funded medical students (*n* = 248).

**Figure 3:**
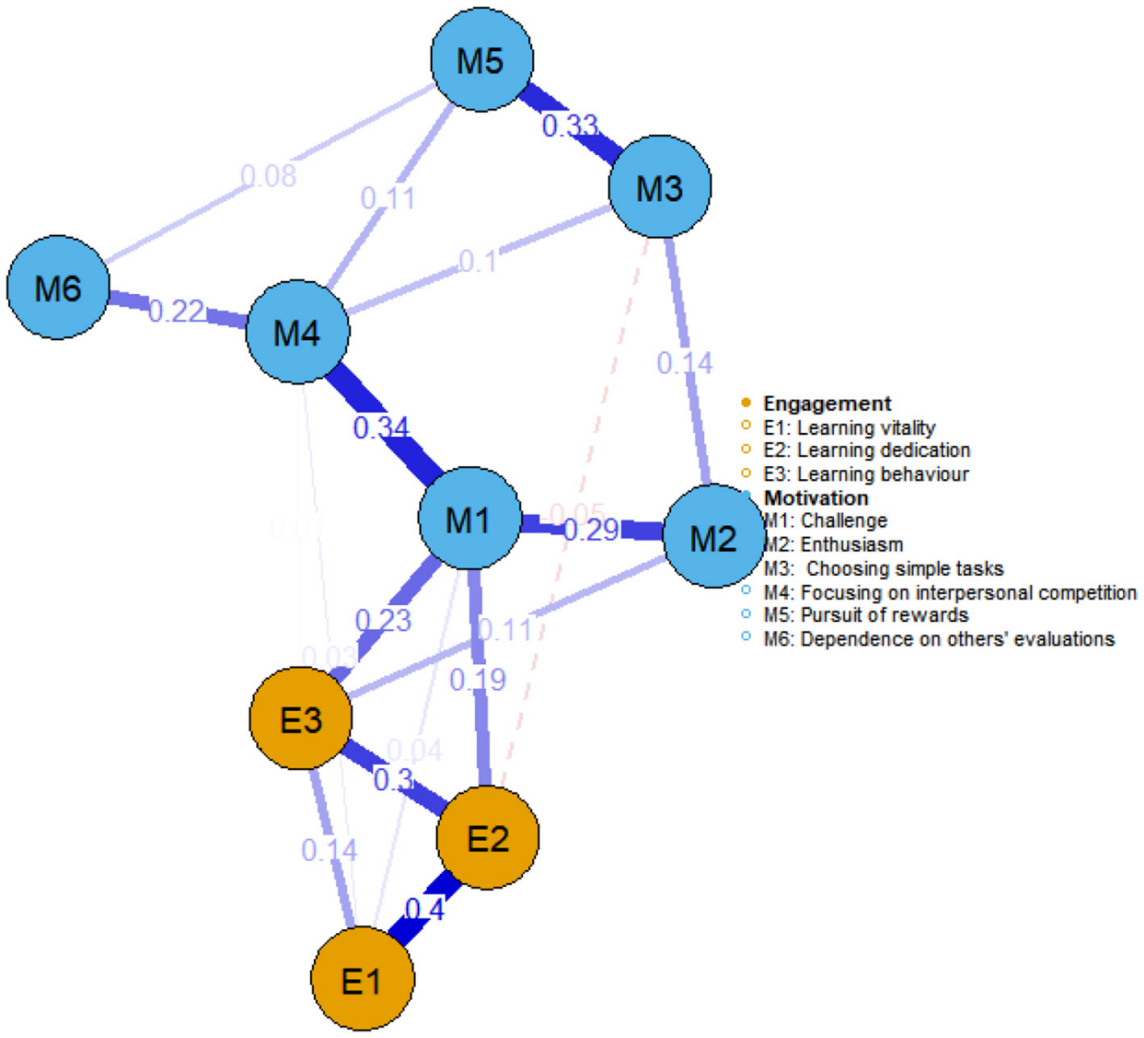
Network structure of learning motivation and learning engagement among self-funded medical students (*n* = 251).

Figures 2, 3 illustrate the network structures of learning motivation and engagement for government-funded and self-funded medical students, respectively. In terms of learning motivation, both groups exhibited a strong association between focusing on interpersonal competition and challenge. In learning engagement, learning dedication and learning vitality also showed a robust connection in both groups. Further analysis revealed differences in the network linkages between motivation and engagement: among government-funded students, focus on interpersonal competition was most tightly connected to learning dedication (weight = 0.21), whereas among self-funded students, the most prominent link was between challenge and learning behavior (weight = 0.23).

### 3.3 Network centrality

**Figure 4:**
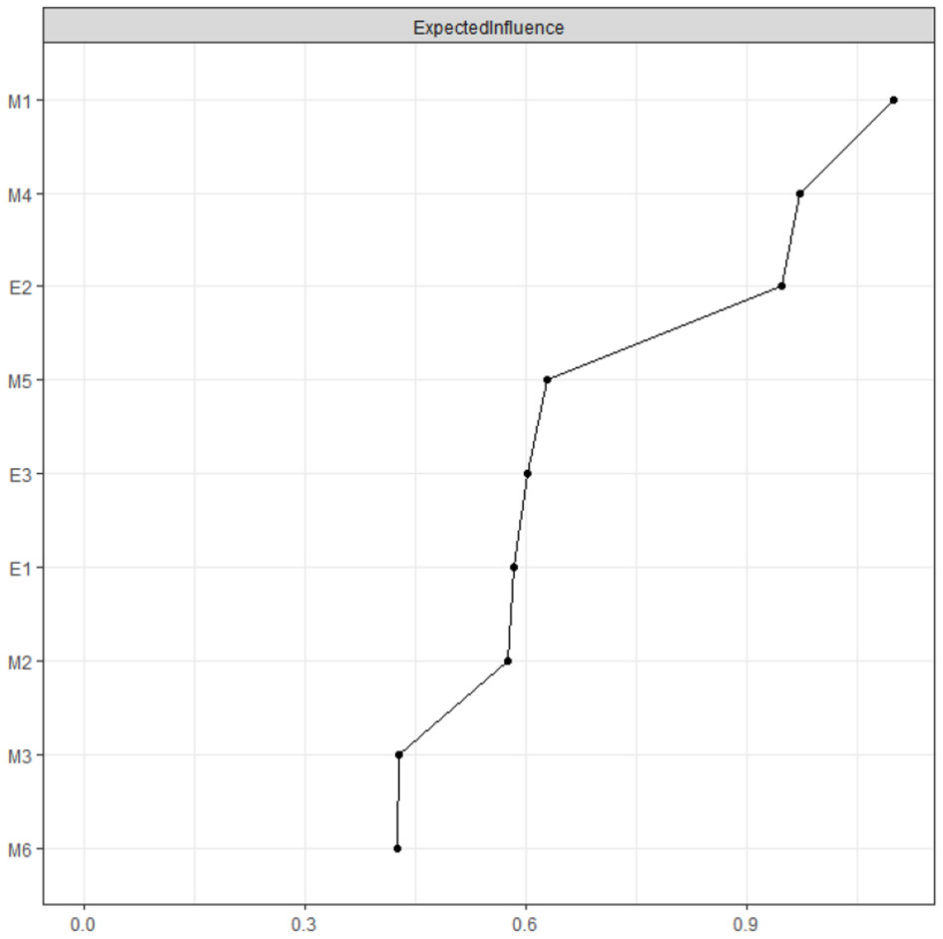
The expected influence of each node in the present network (raw value).

In our network, challenge (intrinsic) and focusing on interpersonal competition (extrinsic) exhibit the highest Expected Influence (EI) (Figure 4). Practically, small, intentional adjustments to these two dimensions are most likely to cascade to multiple engagement behaviors (e.g., dedication, vitality). For example, in a diagnostic-reasoning course, instructors can deploy staged case-based teaching to elicit engagement. Students begin with a patient presenting with acute abdominal pain and must sequentially analyze history, electrocardiogram, and imaging findings. As information unfolds and conflicting data appear (e.g., discrepant test results), the level of challenge escalates. This design fosters intense focus, prompts autonomous study after class, and sustains motivation derived from resolving difficult problems. Dependence on others' evaluations displayed the lowest EI, suggesting a relatively peripheral position in the learning motivation–engagement network with weak associations to other behavioral indicators.

**Figure 5:**
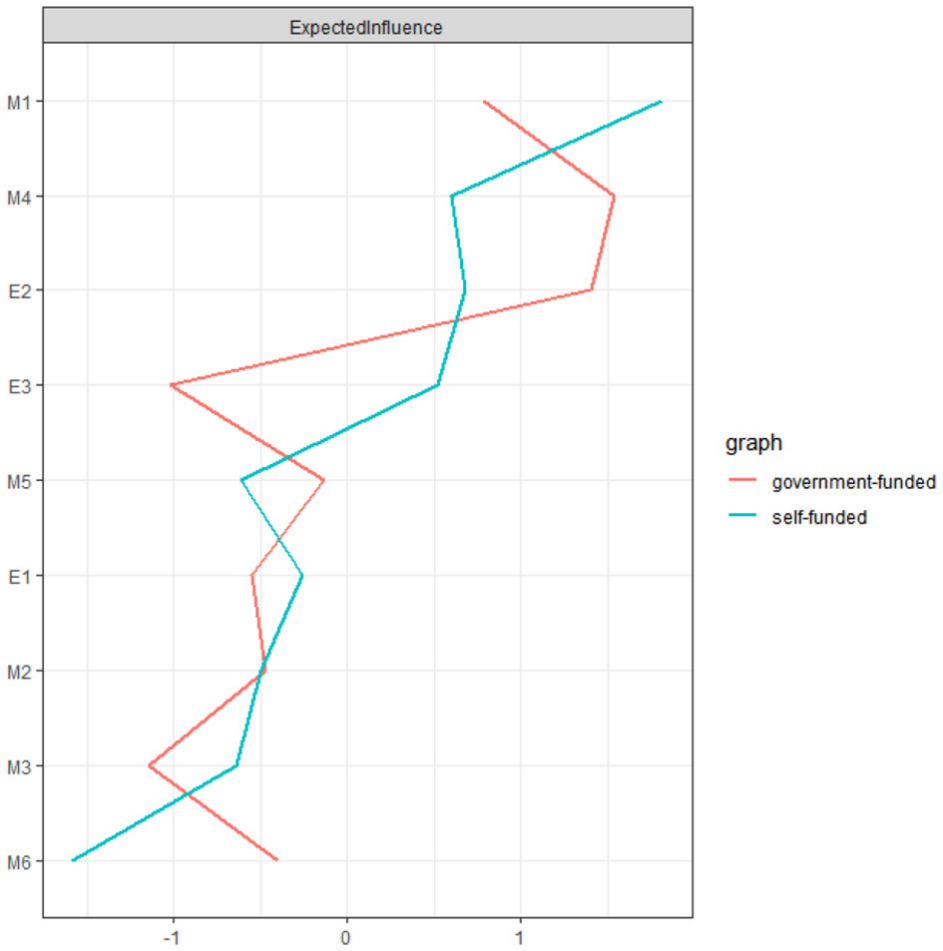
The expected influence of each node in the present network with different training models (blue = self-funded medical students; red = government-funded medical students).

Figure 5 illustrates the EI indices for government-funded and self-funded students. In the government-funded sample, focusing on interpersonal competition ranked the highest, whereas choosing simple tasks showed the lowest EI. Among self-funded students, challenge was the most influential dimension, while dependence on others' evaluations had the lowest EI.

### 3.4 Network accuracy and stability

**Figure 6:**
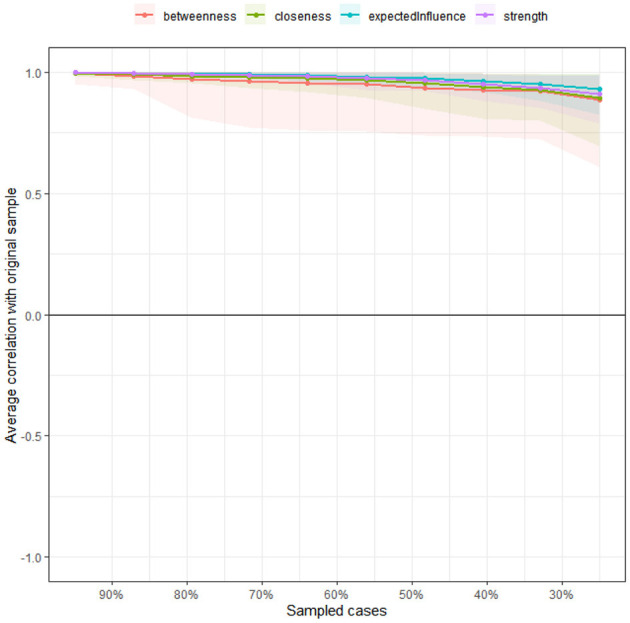
Stability coefficient of node centrality metrics.

**Figure 7:**
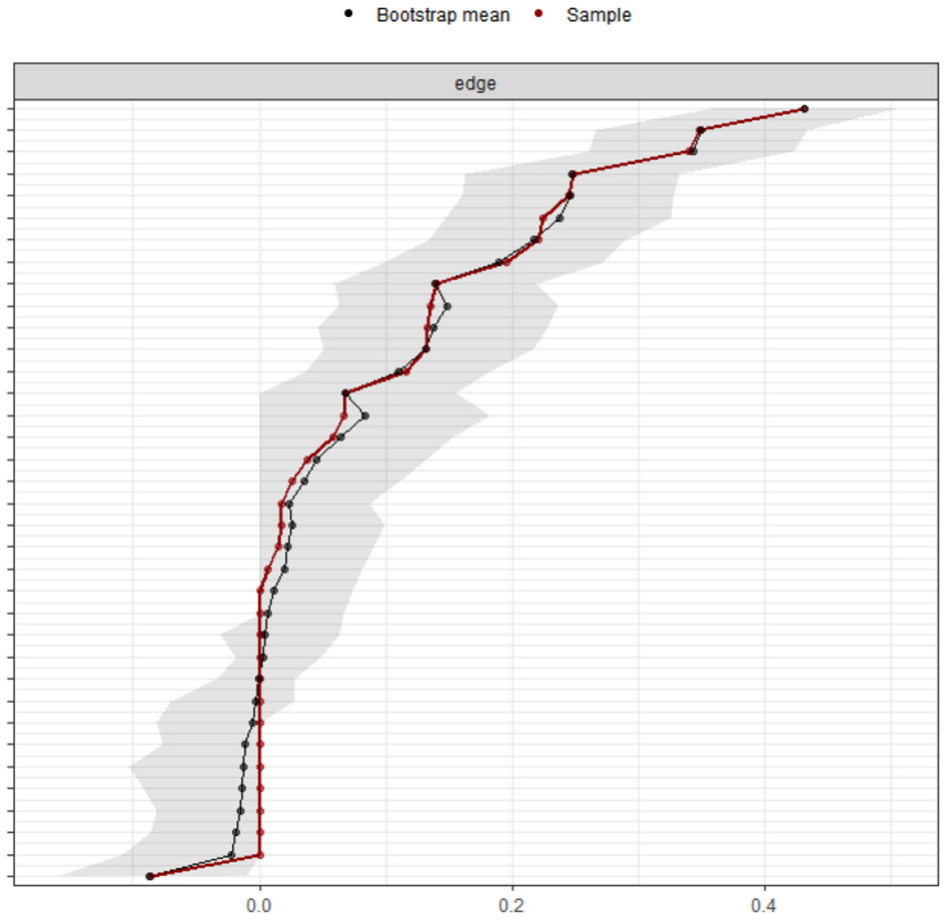
Accuracy of edge weight estimation.

Based on the stability estimates for the centrality metrics (Figure 6) and the bootstrap results of the edge weights (Figure 7), all three networks (overall sample, government-funded students, self-funded students) exhibited high accuracy. The centrality stability (CS) coefficients exceeded 0.75, indicating good overall stability.

Using 95% confidence intervals (CIs) for edge weights to assess network accuracy, the bootstrapped differences in edge weights are shown in Figure 7. The red line indicates edge weights from the original sample, and the black line represents estimates from nonparametric bootstrap methods; the gray area denotes the 95% CI for edge weights. In Figure 7, the red and black lines almost overlap, and the 95% CIs are relatively narrow, suggesting that the estimated edge weights are highly accurate.

### 3.5 Comparisons of network structures between government-funded and self-funded students

Three tests were conducted to compare differences in the learning motivation–engagement networks between government-funded and self-funded medical students. The Network Structure Invariance test showed no significant difference (*p* = 0.10), and the Global Strength Invariance test indicated that the two groups did not differ significantly in overall network strength (*p*= 0.74). Additionally, none of the edges in the Local Invariance test reached statistical significance (*p* > 0.05). These findings suggest a high degree of stability in learning motivation and engagement dimensions across different training models.

## 4 Discussion

### 4.1 Key nodes in the learning motivation and learning engagement network

Our findings indicate that challenge and focusing on interpersonal competition function as central hub nodes in the network, exerting significant influence on the overall structure. When these nodes are activated, their effects spread throughout the network by way of interconnected nodes, thereby shaping the dynamic evolution of the symptom network (Hofmann et al., 2016). As a sub-dimension of intrinsic motivation, challenge emphasizes individual growth and perseverance. Network analyses revealed a strong link between challenge and learning dedication—a dimension of learning engagement—suggesting that these dimensions bridge the learning motivation and engagement sub-networks. This connection may arise because challenging tasks strongly evoke intrinsic motivation, prompting higher levels of engagement among students (Larson and Rusk, 2011). Additionally, such tasks can bolster self-efficacy and a sense of accomplishment, further reinforcing behaviors tied to learning engagement (Shang and Ma, 2024). Within the learning motivation network, challenge is closely linked to both involvement (weight = 0.35) and focusing on interpersonal competition (weight = 0.34). This finding implies that challenge may activate motivation via two pathways: interest-driven involvement and external competitive stimuli.

Prior research has identified an inverted U-shaped relationship between challenge and intrinsic motivation (Ma et al., 2017): motivation increases as challenge level rises until an optimal point, after which motivation begins to decline. Appropriate levels of challenge maximize intrinsic motivation, manifesting in enhanced focus, perseverance, and determination—factors known to significantly improve task performance (Aubé et al., 2014). This has important implications for medical education: when designing learning tasks, educators should account for both the optimal degree of challenge and individual differences. Such precise task design can maximize intrinsic motivation and foster more effective learning and professional growth.

Although the majority of research emphasizes the positive effects of intrinsic motivation on learning engagement, extrinsic motivation also exerts a certain degree of positive influence on learning engagement (Goswami and Urminsky, 2017; Liu et al., 2022). In our study, the focusing on interpersonal competition serves as a pivotal node, significantly impacting the overall network as well. Existing research has explored the relationship between competition and learning engagement from various perspectives. For instance, Xu et al., 2018 empirically found that a healthy competitive attitude can enhance students‘ level of learning engagement, whereas an excessively competitive attitude may diminish their motivation to engage in learning. (Meng et al., 2024) demonstrated a positive correlation between competition and learning engagement. From a positive standpoint, competition can stimulate individuals' intrinsic motivation, prompting learners to focus more intently on learning tasks to gain an advantage in competitive scenarios. Moreover, within a competitive atmosphere, learners are likely to set higher goals and adopt more proactive learning strategies, such as in-depth study of materials, actively seeking feedback, and continuous self-reflection, all of which contribute to improving their academic performance.

According to Maslow's hierarchy of needs (Maslow, 1943), competition may help students recognize their own value and gain respect from others. External feedback is thus internalized into intrinsic motivation, generating sustained positive reinforcement that further promotes competitively oriented learning behaviors. Nevertheless, excessive competition may induce anxiety and stress, ultimately diminishing motivation. Therefore, teaching methods such as problem-based learning (PBL) and case-based learning (CBL) can be adopted to encourage mutual support and teamwork, striking a balance between cooperation and competition.

### 4.2 Comparison of networks between government-funded and self-funded medical students

Comparing networks for publicly funded and self-funded medical students revealed notable differences. Among publicly funded students, interpersonal competition is the most influential node and shows the strongest association with absorption. Instructional design should therefore channel competitive pressure into mastery-oriented collaborative environments. For instance, a redesigned leaderboard can rank not only by position but also recognize accuracy and improvement, and award points for helping peers learn. This preserves the motivational benefits of competition while fostering mutual support and collective progress. During clinical placements, a “buddy-pair” system—two students jointly recording and checking each other's skill gains—can retain the energizing aspects of comparison while curbing unhealthy competition. These strategies maintain the motivational edge of competition yet redirect it toward collective growth, thereby supporting sustained focus and engagement.

For self-funded students, challenge is the central node and is most tightly associated with absorption. Curriculum and assessment can therefore prioritize calibrated difficulty and autonomy-supportive choice. In coursework, progressive-complexity assignments (worked example → partial example → independent case) and choice-based “stretch tasks” invite deeper problem-solving while avoiding overload. In clinical training, short, mentored intensives (e.g., a “mini-fellowship week” on airway management or ultrasound-guided procedures) can consolidate difficult skills, which—per the challenge-dedication- vitality pathways—should propagate to sustained engagement. Reflective difficulty-calibration notes (what was hard, why, how I'll approach it next time) help students internalize adaptive challenge rather than avoid it.

Preference for simple tasks generally reflects a tendency, driven by extrinsic motivation, to choose tasks with clear goals and predictable processes. Our findings show that among government-funded students, preference for simple tasks had the lowest expected influence index, suggesting that it exerts a relatively weak effect on learning behavior in this group. Faced with high levels of competitive pressure, these students may actively opt for more difficult tasks to improve their capabilities and competitiveness, rendering simple tasks insufficiently challenging to spark motivation. Meanwhile, among self-funded students, reliance on others' evaluations exhibited the lowest expected influence index, potentially for two reasons: first, such students tend to be more autonomous and independent, preferring to evaluate their own performance and set personal standards; second, many self-funded students chose the medical profession out of personal interest, resulting in a stronger intrinsic drive and less dependence on external validation. From an intervention perspective, therefore, educators of government-funded students might focus on stimulating intrinsic motivation by providing more challenging, practice-oriented tasks that meet their needs for skill enhancement. For self-funded students, strategies should emphasize fostering autonomy and self-reflection, encouraging learning plans driven by personal interests and internal goals rather than external feedback.

## 5 Limitations

By quantifying each network node's influence and linkage patterns, this study highlights the complexity of learning motivation in medical education. Identifying core dimensions offers practical guidance for curriculum design (e.g., incorporating challenging tasks to activate hub nodes) and underscores the need to tailor motivational strategies to different student groups. Future research could investigate the longitudinal dynamics of the network to assess the long-term effects of educational interventions on reshaping network structures. Nonetheless, several limitations must be acknowledged. First, the network model in this study was developed solely from self-reported measures of learning motivation and engagement, raising the possibility of subjective bias. Subsequent research may adopt diverse data collection methods, such as observational and interview-based approaches, for more comprehensive and objective assessments. Second, the cross-sectional design limits inferences about dynamic causal relationships among variables. Longitudinal studies employing network analyses could further clarify the temporal changes in learning motivation and engagement. Lastly, because the sample was drawn from a single medical university, generalizability may be limited. Future research could expand the sample size and adopt national, multi-center, longitudinal data to capture a more complete picture of the network structure and its evolution.

## 6 Conclusion

Unlike traditional approaches that aggregate dimensions or impose single-path models, psychological network analysis maps conditional dependencies, preserves the sign of associations, and pinpoints central/bridge nodes (e.g., challenge, interpersonal competition) with high expected influence—direct levers for intervention. It also enables formal tests of structural and strength invariance across training models, revealing stable yet interpretable differences that bivariate correlations or omnibus regression would likely obscure. This network analysis of 499 medical students' learning motivation and engagement revealed that challenge had the highest expected influence index, followed by focus on interpersonal competition; these two dimensions acted as central hubs exerting significant effects on the overall network. Comparative analyses indicated that government-funded students' engagement was largely driven by extrinsic motivation, whereas self-funded students were chiefly influenced by intrinsic motivation. Building on these findings, we recommend that, at the level of instructional practice, curriculum design emphasize graduated difficulty and mechanisms that support autonomous choice; assessment systems incorporate constructive competitive tasks and scoring that captures individual growth; and, in clinical training, programs integrate mentor-guided high-difficulty rotations and team-based performance benchmarks. Future research should employ longitudinal designs or temporal network analyses to test whether interventions targeting challenge and interpersonal competition produce the expected downstream changes in engagement nodes; extend to multicenter cohort sampling; and integrate self-report with behavioral/learning-trace data to yield stronger inferences.

## Data Availability

The raw data supporting the conclusions of this article will be made available by the authors, without undue reservation.
